# Global co-expression network for key factor selection on environmental stress RNA-seq dataset in *Capsicum annuum*

**DOI:** 10.1038/s41597-023-02592-3

**Published:** 2023-10-12

**Authors:** Junesung Lee, Seon-In Yeom

**Affiliations:** Division of Applied Life Science (BK21 Four), Institute of Agriculture & Life Science, Gyeongsang National University, Jinju, 52828 Korea

**Keywords:** Plant stress responses, Plant immunity, Plant signalling

## Abstract

Environmental stresses significantly affect plant growth, development, and productivity. Therefore, a deeper understanding of the underlying stress responses at the molecular level is needed. In this study, to identify critical genetic factors associated with environmental stress responses, the entire 737.3 Gb clean RNA-seq dataset across abiotic, biotic stress, and phytohormone conditions in *Capsicum annuum* was used to perform individual differentially expressed gene analysis and to construct gene co-expression networks for each stress condition. Subsequently, gene networks were reconstructed around transcription factors to identify critical factors involved in the stress responses, including the NLR gene family, previously implicated in resistance. The abiotic and biotic stress networks comprise 233 and 597 hubs respectively, with 10 and 89 NLRs. Each gene within the NLR groups in the network exhibited substantial expression to particular stresses. The integrated analysis strategy of the transcriptome network revealed potential key genes for complex environmental conditions. Together, this could provide important clues to uncover novel key factors using high-throughput transcriptome data in other species as well as plants.

## Introduction

Environmental stress is a major challenge for plants as it affects their growth, development, and productivity^1,2^. Plants encounter various types of stress, including abiotic factors, such as drought, heat, and salinity, as well as biotic factors, such as pathogen attacks and insect infestations^3^. These stressors induce complex molecular responses in plants leading to changes in gene expression, signaling pathways, and physiological adaptations^4,5^. Multiple studies have been conducted to identify genes involved in responses to diverse environmental stressors and one of the primary approaches utilizes transcriptome data for large-scale data analysis.

RNA-seq is one of the main methods used to generate transcriptome data and has been used successfully in numerous studies to discover key genetic factors^6^. Along with RNA-seq, differential expression gene (DEG) analysis is a powerful approach to highlight gene expression changes. DEG has been widely used to identify key genes involved in responses to different treatments^7^. Gene expression patterns can also be utilized to analyze gene-gene interactions and to identify key regulatory factors in pathways and gene clusters^8^.

DEG-based gene co-expression networks (GCNs) play crucial roles in understanding the complex regulatory mechanisms underlying biological systems. These networks capture the relationships among genes based on their expression patterns under various conditions and/or in various tissues. By analyzing the co-expression patterns of genes, researchers gain insights into functional modules, regulatory pathways, and disease mechanisms^9^. In pepper, key resistance genes were identified successfully through GCN analysis, which proved to be effective for identifying core regulators among the entire pool of resistance genes^10^. However, research that analyzes complex stress-based networks to identify key genetic factors are lacking.

In this study, we introduce a method to identify key genes using GCNs based on RNA-seq data. By integrating and reconstructing individual networks around specific functions, this approach builds on the foundation of conventional RNA-seq analysis by placing greater emphasis on GCN construction. To demonstrate the efficiency of this method, we analyzed RNA-seq data from the pepper under environmental stress conditions^11–13^. Through this analysis process, we identified key regulatory genes that form the hub of the network and demonstrated the efficiency of the analysis method by identifying defense response genes that are highly expressed in the stress environment^14,15^.

## Results

### Strategies for data analysis

**Fig. 1:**
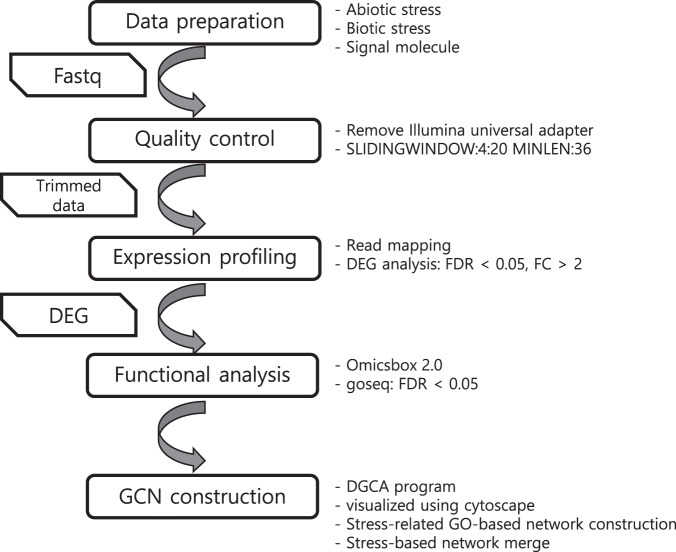
The GCN construction strategy. The methods used for each step are listed to the right of the step. For further details, please see the Methods section of the manuscript.

This study uses a research methodology that involves constructing a network from RNA-seq data to identify regulators associated with the desired phenotype. Genes identified through this process are expected to play important roles. The dataset used in this study included abiotic and biotic stressors as well as phytohormone conditions. The procedural framework is shown in Fig. 1. The overall workflow includes data acquisition, quality control, expression profiling, functional analysis, and sequential construction of a GCN.

### Data preprocessing and transcriptome data variation analysis

**Fig. 2:**
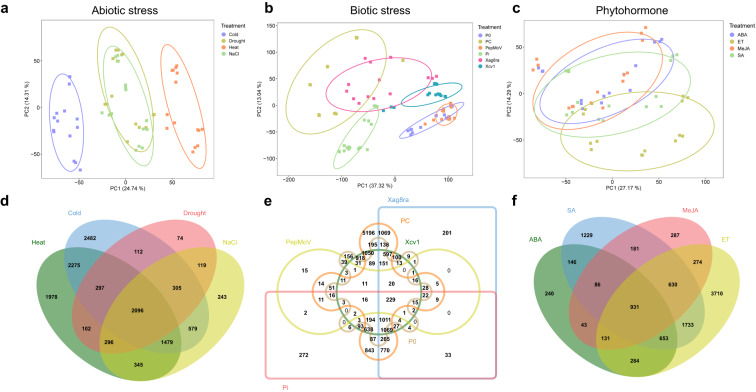
Principal components analysis (PCA) and differential expression gene (DEG) analysis under abiotic, biotic, and phytohormone stress conditions. PCA scatter plots of the abiotic stress condition (**a**), the biotic stress condition (**b**), and the phytohormone stress condition (**c**). DEG-based Venn diagrams of the abiotic stress condition (**d**), the biotic stress condition (**e**), and the phytohormone stress condition (**f**).

We obtained abiotic (204.7 Gb), biotic (344.6 Gb), and phytohormone (188 Gb) datasets from the NCBI Sequence Read Archive database (Table 1). Each dataset was processed to obtain 637.6 Gb after the removal of adapter sequences using the Cutadapt program and quality trimming. These data were used to perform read mapping and the mapped reads were normalized to fragments per kilobase of transcript per million mapped reads (FPKM) for inter-sample comparisons. Principal component analysis (PCA) was performed for each stress group to assess the variation between samples. In addition, DEG analysis was performed to verify data integrity. In the abiotic stress dataset, the heat and cold stress samples formed separate clusters, whereas the drought and salinity stress samples were grouped together (Fig. 2a). In the biotic stress dataset, each stress condition formed a distinct cluster (Fig. 2b). In the phytohormone dataset, except for the ethylene (ET) treatment, the treatments showed similar trends at early time points but showed significant variation at later time points (Fig. 2c).

**Table 1:** Transcriptome characteristics.

Treatment	Cleaned data (Gb)	Number of DEGs	Number of nodes	Number of edges	Accession numbers
**Abiotic stress**					
Cold	38.1	9,626	7,664	1,922,716	SRP187794
Salinity (NaCl)	43.2	5,463	4,302	145,024	SRP187794
Heat	34.3	8,869	6,309	270,163	SRP187794
Drought (Man)	41.0	3,402	2,571	78,221	SRP187794
**Biotic stress**					
*P. capsici* (Pc)^a^	33.1	13,838	11,369	2,686,735	SRP438321
*P. infestans* (Pi)^b^	27.0	5,648	3,954	223,552	SRP106410
TMV_P0 (P0)^c^	6.3	2,792	2,319	87,686	SRP119199
PepMoV^d^	9.9	545	383	4,672	SRP119199
Xcv1^e^	85.3	6,320	5,276	1,084,241	SRP438321
Xag8ra^f^	11.6	5,809	5,068	255,855	SRP438321
**Phytohormones**					
ABA (abscisic acid)	40.4	2,515	1,766	49,994	SRP265260
ET (ethylene)	38.5	8,347	5,648	267,252	SRP265260
MeJA	34.6	2,564	1,910	39,738	SRP265260
SA (salicylic acid)	32.8	5,590	3,847	213,586	SRP265260

^a^*Phytophthora capsici.*

^b^*Phytophthora infestans.*

^c^Tobacco mosaic virus.

^d^Pepper mottle virus.

^e^*Xanthomonas campestris pv. Vesicatoria race 1.*

^f^*Xanthomonas axonopodis pv. glycines 8ra.*

### DEG-based GCN construction

To investigate changes in gene expression, we compared the expression levels at each time point to the corresponding mock condition. Among the samples, the *Phytophthora capsici* (Pc)*-*treated group exhibited the highest number of differentially expressed genes (DEGs) with 13,838 genes identified, whereas the pepper mottle virus (PepMoV)-treated group had the fewest DEGs with 545 genes (Table 1). We identified 12,782, 15,774, and 11,558 DEGs in the abiotic, biotic, and phytohormone groups, respectively, all of which met the criteria of log_2_FC > |1| and FDR < 0.05.

**Fig. 3:**
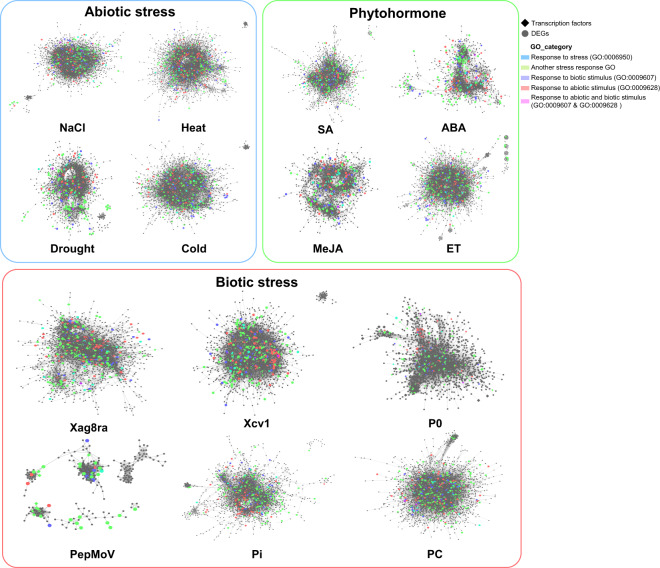
Gene expression networks in response to stress conditions. We constructed individual networks based on stress-responsive differentially expressed genes (DEGs). Each node in a network represents either a transcription factor (TF) or a non-TF gene. The shape of the node indicates whether it represents a TF or a non-TF gene. Additionally, we classified genes into different categories based on gene ontology (GO) categories and assigned colors to the nodes accordingly.

Across the abiotic stress groups, 2,096 DEGs were commonly identified. The cold stress-specific group had the highest number of DEGs with 2,482 genes (Fig. 2d). In the biotic stress group, there were 5,196 Pc-specific DEGs, and 229 genes were commonly identified across all six biotic stress conditions (Fig. 2e). Regarding phytohormones, 931 genes were commonly identified, and there were 3,710 ET-specific DEGs (Fig. 2f). Furthermore, we conducted GCN analysis using the DEGs to identify potential hub genes that interact with other genes and key regulatory modules associated with the stressors. The GCN analysis revealed connected gene modules with coordinated expression patterns indicating their interactions during the stress responses (Fig. 3). We constructed 14 networks using 345 samples and the network with the highest number of nodes was in the Pc network, which consisted of 11,369 nodes. This network revealed the most extensive connectivity with a total of 2,686,735 edges, whereas the average network had 4,456 nodes and 523,531 edges.

### Building an integrated network with gene set enrichment analysis (GSEA)

**Fig. 4:**
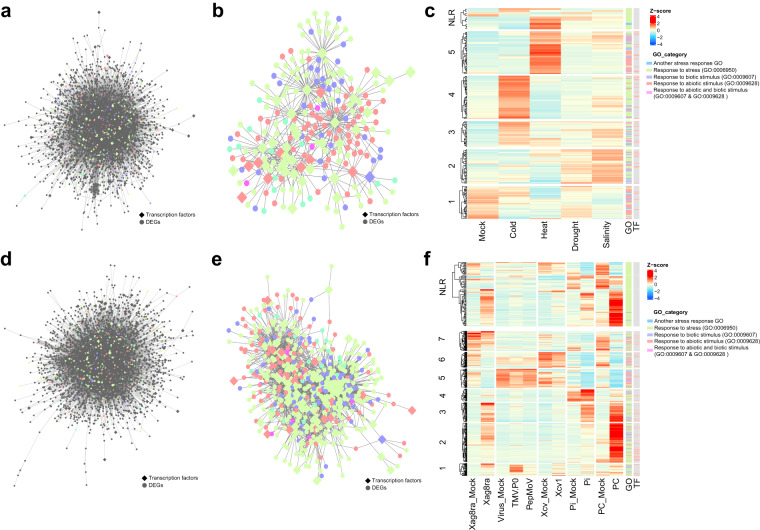
Identification of key transcription factors through integrated network reconstruction. We performed integrated network reconstruction to identify key transcription factors (TFs) using individual networks divided into two groups: abiotic and biotic stress. The integrated abiotic stress network (**a**) and the integrated biotic stress network (**d**) were constructed with diamond-shaped nodes representing TFs and circle-shaped nodes representing non-TF DEGs. Subsequently, we reconstructed abiotic (**b**) and biotic (**e**) networks based on GO categories to investigate interactions between stress-related TFs and DEGs. To compare expression patterns of genes, we constructed Z-score-based heatmaps for abiotic stress conditions (**c**) and biotic stress conditions (**f**). In the TF column, TF genes are indicated in red.

We constructed an integrated network by merging networks based on DEGs to identify key regulatory genes associated with abiotic and biotic stressors. The abiotic stress-integrated network consisted of 10,881 nodes and 2,365,260 edges (Fig. 4a), whereas the biotic stress-integrated network consisted of 13,290 nodes and 4,149,084 edges (Fig. 4d). Each network was functionally classified based on stress-related genes. Subsequently, hub genes were selected by focusing on transcription factors (TFs) in each network to identify key interacting genes. These core networks were reconstructed based on stress-related gene ontologies (GOs), and the reconstructed abiotic stress network consisted of 233 nodes and 443 edges, and the reconstructed biotic stress network consisted of 597 nodes and 3,323 edges (Fig. 4b,e). From these networks, we were able to classify interactions between stress-related genes that are strongly regulated by stress-related TFs.

We also performed a heatmap analysis to infer gene function and examine expression patterns within each network. In the abiotic stress heatmap, genes were grouped into five clusters. Cluster 1 included genes with strong expression in the mock condition, but reduced expression under heat stress. Cluster 2 included genes with strong expression under salinity and drought stress conditions. Clusters 3 and 4 included genes with strong expression under cold stress, and cluster 5 included genes with strong expression under heat stress. Interestingly, many genes with strong expression under heat stress were in the nucleotide binding and leucine-rich repeat (NLR) gene group, which is a major part of the disease-resistance gene family (Fig. 4c).

Similarly, in the biotic stress environment, genes were grouped into eight clusters. Cluster 2 had the highest number of genes and the most pronounced changes in gene expression in the presence of Pc. We also observed a strong expression of NLR genes in the presence of Pc (Fig. 4f). Based on these results, genes in the networks associated with stress are likely to perform resistance functions. In addition, the presence of numerous NLR genes, which are known for their resistance functions, confirms the usefulness of this analysis to identify key genetic factors across different studies.

## Discussion

This study presents a novel and effective approach that utilizes high-throughput RNA-seq data to identify key genetic factors involved in plant responses to environmental stress. Environmental stressors pose significant challenges to plants by affecting their growth, development, and productivity. To overcome these challenges and develop stress-tolerant crops, a deeper understanding of the underlying molecular responses is essential.

In this study, we used RNA-seq data to analyze gene expression changes in response to different environmental stressors, including abiotic stress, biotic stress, and phytohormone treatments. Stressors induce complex molecular responses in plants leading to changes in gene expression, signaling pathways, and physiological adaptations. We used three primary analytical approaches: (1) DEG analysis, (2) GCN construction, and (3) integrated network construction. DEG analysis is a powerful method to identify genes that change expression significantly under specific stress conditions. By comparing gene expression levels between stress-treated and control samples, a significant number of DEGs associated with each stress condition were identified.

Integration of the DEG-based GCNs plays a crucial role in understanding the complex regulatory mechanisms underlying biological systems. These networks capture the relationships between genes based on their expression patterns under different conditions and/or in different tissues. By analyzing the co-expression patterns of genes, researchers can gain important insights into functional modules, regulatory pathways, and disease mechanisms. The results of this study demonstrated the successful construction of GCNs for each stress condition and highlighted the interplay between stress-responsive genes and potential regulatory factors. The networks provided valuable information on gene interactions and revealed key regulators in stress responses.

In addition, the integrated network approach proved particularly valuable because it allowed abiotic and biotic stress networks to be merged and allowed the identification of key interacting genes regulated by stress-related TFs. Thus, a comprehensive view of stress-responsive gene networks was formed that led to the identification of core regulatory modules associated with stress responses. Network analysis has been used successfully in several studies to identify gene clusters of interest, for example, in the pepper, it was used to identify key genes within the RLP gene family^10^. In this study, using an integrated network construction approach, we successfully identified TFs and NLR genes that respond to each stress condition confirming the acquisition of these key factors in the pepper^16^. In previous reports, NLR genes were found to be responsive to abiotic and biotic stresses, and the results of the present study also showed strong changes in their expression^17–19^. This research method proves to be a valuable tool for obtaining robust candidate resistance genes because it can be used effectively to identify important resistance-related genes.

In conclusion, this study presents a comprehensive and systematic approach to identifying key genetic factors associated with plant responses to environmental stress. The integration of high-throughput RNA-seq data, DEG analysis, and GCNs provides valuable insights into stress-responsive gene interactions and regulatory mechanisms. The results will contribute to our understanding of plant stress responses and have the potential to advance the development of stress-tolerant crops to meet the challenges posed by environmental stressors in agriculture.

## Methods

### Description of the RNA-seq data used in this study

This study was conducted to select genes that respond to abiotic and biotic stresses using stress response RNA-seq to validate the efficiency of the assay. Responses four abiotic stressors (cold, salinity, drought, and heat), two oomycete pathogens (*P. capsici* and *P. infestans*), two viruses (PepMoV and TMV-P0), two bacteria (Xcv1 and Xag8ra), and four phytohormones (ABA, ET, MeJA, and SA) were compared to determine the response of different environments (Table 1). All RNA-seq data were acquired from the NCBI Sequence Read Archive: abiotic stress (SRP187794^20^), biotic stress (SRP106410^21^, SRP119199^22^, SRP438321^23^), signal molecule (SRP265260^24^). The gene expression patterns used in this study were obtained from the information registered in the Gene Expression Omnibus (GEO) of the NCBI (GSE149037^25^, GSE132824^26^, and GSE240234^27^).

### Data preprocessing and quality control

In the read quality control step of this study, adapter trimming was performed using Cutadapt^28^, and reads with Phred scores below 20 were filtered out using Trimmomatic^29^. To remove residual adapter sequences from the reads, Cutadapt was used to specifically target the Illumina universal adapter, which is used widely in Illumina sequencing to facilitate proper attachment of sequencing primers to DNA fragments. For further quality control, Trimmomatic was utilized to filter out reads with low-quality bases, and the following parameters were applied during this step: LEADING: 3, TRAILING: 3, SLIDINGWINDOW: 4: 20, and MINLEN: 36. These settings ensured that any read with a Phred score below 20 (indicating a base call accuracy of 99%) would be excluded from subsequent analysis. For read alignment, HISAT2^30^ was employed using default parameters. The reference genome assembly used was the *Capsicum annuum* 2.0 version which was downloaded from PGENOME (http://peppergenome.snu.ac.kr).

### Differential expression gene (DEG) analysis

Transcript abundance was quantified using fragments per kilobase of transcript per million mapped reads (FPKM). Quantification was performed manually. DEG analysis was performed using the DESeq2 package^31^ in R. The following criteria were used to identify differentially expressed genes (DEGs): genes with an adjusted *p*-value (FDR) less than 0.05 and a log_2_fold change (log_2_FC) greater than |2|. DEGs were analyzed further for their functional implications.

### Co-expression network construction and visualization

Based on the DEGs, we constructed a co-expression network using the DGCA R package^32^. DGCA evaluates interactions between genes based on co-expression patterns and forms a network structure. The network was constructed based on gene pairs with a correlation of 0.9 or more, and the TFs and GOs of the nodes in each connection were classified^10^. The co-expression network generated by DGCA was visualized using Cytoscape^33^. We constructed an integrated network by focusing on the genes shared within individual stress networks. The network was then reconstructed around the TFs for core network analysis, and direct correlation genes were obtained around the hub gene for analysis.

### Functional annotation and enrichment analysis

In this study, OmicsBox 2.0 (https://www.biobam.com/omicsbox/) was used to functionally annotate the identified DEGs. OmicsBox 2.0 matched DEGs with available databases to retrieve functional annotations based on sequence homology and other relevant information. To further explore the functional implications of the DEGs, enrichment analysis was conducted using the goseq package^34^ in R. The goseq package incorporates the gene length bias inherent in RNA-seq data and performs gene ontology (GO) enrichment analysis. We focused on the stress-related gene ontology (GO: 50896) category to investigate genes associated with stress responses and adaptation. We reconfigured the integrated networks by focusing on the abiotic and biotic stress-related GO terms. We then performed expression profiling based on genes associated with stress-related GO terms. Heatmap analysis was performed to evaluate gene expression within a network. The expression levels of each gene were assessed using Z-scores to determine differences between treatment groups. To normalize expression levels based on treatment conditions, average values were calculated for each time point. This normalization allowed for comparative analyses of gene expression patterns across different treatments. For visualization, we used the ComplexHeatmap R package^35^.

## Data Availability

All the data generated in this study has been uploaded to Figshare (10.6084/m9.figshare.23659218.v2)^36^. The data has been organized into the following categories: Z-score, DEG, gene ontology, gene correlation information, and network node/edge information. Detailed information can be found in the description within the Figshare.

## Code availability

The R code used in this study is provided at Figshare and Github (https://github.com/Lee-June-Sung/Co-expression_network_using_DGCA.git).
